# Pirfenidone Protects from UVB-Induced Photodamage in Hairless Mice

**DOI:** 10.3390/molecules28072929

**Published:** 2023-03-24

**Authors:** Yocasta Martinez-Alvarado, Eduardo Amezcua-Galvez, Judith Davila-Rodriguez, Ana Sandoval-Rodriguez, Marina Galicia-Moreno, Mónica Almeida-López, Silvia Lucano-Landeros, Arturo Santos, Hugo Christian Monroy-Ramirez, Juan Armendariz-Borunda

**Affiliations:** 1 Programa de Doctorado en Ciencias en Biología Molecular en Medicina, CUCS, University of Guadalajara, Guadalajara 44340, Mexico; 2 Department of Molecular Biology and Genomics, Institute of Molecular Biology in Medicine and Gene Therapy, CUCS, University of Guadalajara, Guadalajara 44340, Mexico; 3 Department of Dermatology, Civil Hospital of Guadalajara “Fray Antonio Alcalde”, Guadalajara 44280, Mexico; 4 Department of Pathology, Civil Hospital of Guadalajara “Fray Antonio Alcalde”, Guadalajara 44280, Mexico; 5 Department of Basic Psychology, CUCS, University of Guadalajara, Guadalajara 44340, Mexico; 6 School of Medicine and Health Sciences, Tecnologico de Monterrey, Zapopan 45138, Mexico

**Keywords:** photodamage, pro-inflammatory markers, ultraviolet radiation, skin, pirfenidone

## Abstract

Background: Ultraviolet radiation (UV) is the main environmental factor that causes histological degenerative changes of the skin giving rise to a chronic process called photodamage. Non-melanoma skin cancer induced by UVB radiation is a result of a cascade of molecular events caused by DNA damage in epidermis cells, including persistent inflammation, oxidative stress, and suppression of T cell-mediated immunity. Retinoids such as tretinoin have been widely used in skin to treat photoaging and photodamage, though its secondary adverse effects have been recognized. Pirfenidone (PFD) has emerged as an antifibrogenic, anti-inflammatory and antioxidant agent, and in this work its efficacy was evaluated in a model of UVB-induced photodamage. Methods: Epidermal, dermal, and inflammatory changes were measured by histomorphometric parameters. In addition, gene, and protein expression of key molecules in these processes were evaluated. Results: Our results revealed an anti-photodamage effect of topical PFD with absence of inflammatory skin lesions determined by dermoscopy. In addition, PFD reduced elastosis, improved organization, arrangement, and deposition of dermal collagens, downregulated several pro-inflammatory markers such as NF-kB, IL-1, IL-6 and TNFα, and decreased keratinocyte damage. Conclusion: Topical pirfenidone represents a promising agent for the treatment of cell photodamage in humans. Clinical trials need to be carried out to explore this premise.

## 1. Introduction

Direct environmental skin damage due to UV radiation exposure causes accumulative cellular and molecular changes in skin layers associated with photoaging and skin cancer [1,2]. Although UVB radiation is only 0.3% of solar emission, it is the main cause of sunburn and skin cancer [3]. UVB photodamaged skin shows pigmentation, inflammatory cell infiltrate, epidermal thickness, and dermal proteins oxidation; eventually leading to oxidative stress, immunosuppression, photoaging, and photo-carcinogenesis [4]. UVB also causes cellular changes including atypia of epidermal keratinocytes and melanocytes, loss of Langerhans cells, and flattening of the dermo epidermal junction [5,6]. Dermal alterations include increased/fragmented procollagen I and III [7], accumulation and displacement of elastic fibers into the deep dermis, known as elastosis, which is a photoaging hallmark [8]. UV radiation can activate cell surface receptors and intracellular signals mediated by kinases, promoting nuclear transcription complex activator protein 1 (AP-1) activation, even in sub erythemal UVB dose [9,10,11]. Moreover, in skin, TGF-β1 regulates keratinocytes growth and differentiation, and increases proinflammatory cytokines production (IL-1, IL-2, TNF-α, and INF-γ) induce an inflammatory stage that promotes tumors formation or accelerates neoplasm growth, and finally carcinoma conversion [12,13]. In addition, UVB activates nuclear factor-κB (NF-κB) [6,14]. Inflammatory cells infiltration plays an important role in tissue damage and dermal proteins degradation by metalloproteinases (MMPs), whose main source of secretion is neutrophils infiltrate. [15,16]. Persistently, these events trigger a suitable environment for photoaging and skin cancer. 

Tretinoin (TRE) has been used in the dermatologic field for 40 years and is currently approved for acne vulgaris and photodamage treatment [17]. However, its use is associated with a poor tolerability, skin dryness, tightness, erythema, and heightened sunburn potential [18,19]. TRE improves stratum corneum compaction, melanin granules dispersion, epidermal hyperplasia, and synthesis of collagen, elastin, and fibronectin [20,21,22,23]. In addition, it reverses photoaging blocking dermal matrix degradation, hindering the induction of AP-1 and MMPs [24]. However, SKH-1 mice radiated with simulated sunlight, UVA or UVB, and treated with TRE 0.001% showed severe irritation and an increase in the number of skin tumors [25]. These adverse events have driven the development of new retinoids and retinoid-analogs, as well as slow-release TRE formulations. 

PFD is a pyridine (1-Phenyl-5-Methyl-2-(1H)-pyridone) that has shown an adequate security in doses up to 2500 mg orally per day [26]. PDF has been extensively used to treat idiopathic pulmonary fibrosis [27,28]. In liver fibrosis it has shown anti-inflammatory and anti-fibrogenic effects [29,30]. PFD has also been used with good results in the hypertrophic scars treatment secondary to burns and chronic diabetic foot ulcers [31,32]. In addition, its use in the treatment of localized scleroderma improves skin lesions [33]. The aim of this work was to evaluate PFD efficacy to prevent experimental photodamage and study the molecular pathways involved. Our results demonstrated that PFD administration is safe when applied topically and might be used as a novel alternative to treat photodamage and prevent skin cancer development.

## 2. Results

### 2.1. PFD Prevents Photodamage in Hairless Mice Exposed to UVB Radiation

**Figure 1:**
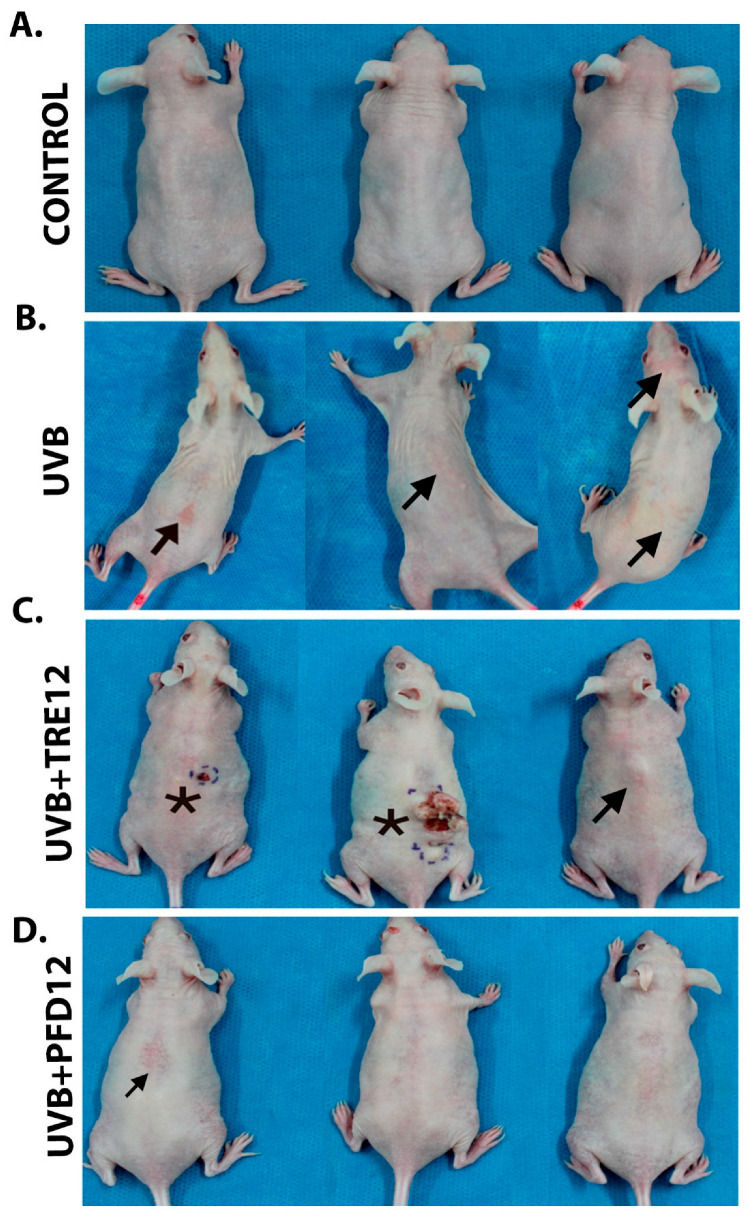
(**A**) Control group mice, animals not exposed to UVB radiation. (**B**) UVB group, mice exposed to UVB radiation, exploration of the dorsal area shows severe inflammatory lesions (→). (**C**) UVB + TRE12 group, mice treated with TRE (0.05% Retin-A^®^ cream) after UVB irradiation, tumor lesions (*) and inflammatory lesions (→) are observed. (**D**) UVB + PFD group, mice treated with PFD (8% gel) after UVB irradiation, only mild inflammation is observed.

**Figure 2:**
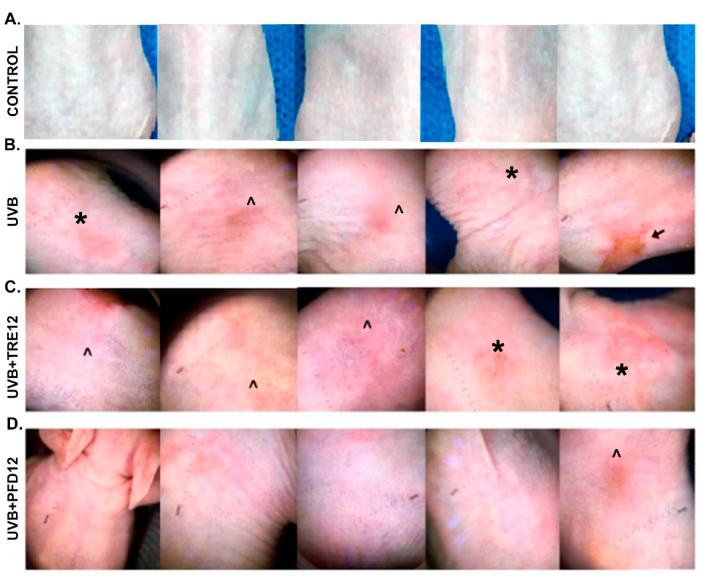
Dermoscopic analysis of skin lesions. (**A**) Group of control mice, without exposure to UVB irradiation. (**B**) UVB group, mice exposed to UVB irradiation, examination of the dorsal area revealed inflammatory skin lesions such as macules (*) and ulceration (↑); and non-inflammatory skin lesions such as scales (^). (**C**) UVB + TRE12 group, mice treated with TRE (0.05% Retin-A^®^ cream) inflammatory skin lesions such as macules (*) and ulceration (↑) are also observed; and non-inflammatory skin lesions such as scales (^). (**D**) UVB + PFD group, mice treated with PFD (8% gel), in which only few, non-inflammatory, scale-like skin lesions were developed (^).

Macroscopic evaluation by photography and dermoscopy in UVB-exposed mice indicated the presence of wrinkles on dorsal skin, hyperpigmented macules, and several inflammatory skin lesions induced by UVB radiation (Figure 1 and Figure 2). Papules, erythema, and ulcers were observed in UVB and UVB + TRE12 groups as studied by dermoscopy (Figure 2A,B). Noteworthy, UVB + TRE12 induced the development of skin tumors (Figure 1C). On the other hand, UVB + PFD12 treated mice showed almost normal appearance of the skin, winkle formation was ameliorated, and lesions were repaired by the treatment as seen by dermoscopy (Figure 2C). 

### 2.2. PFD Was Effective to Prevent Photodamage-Induced Epidermal and Dermal Skin Changes in Hairless Mice Exposed to UVB Radiation

**Figure 3:**
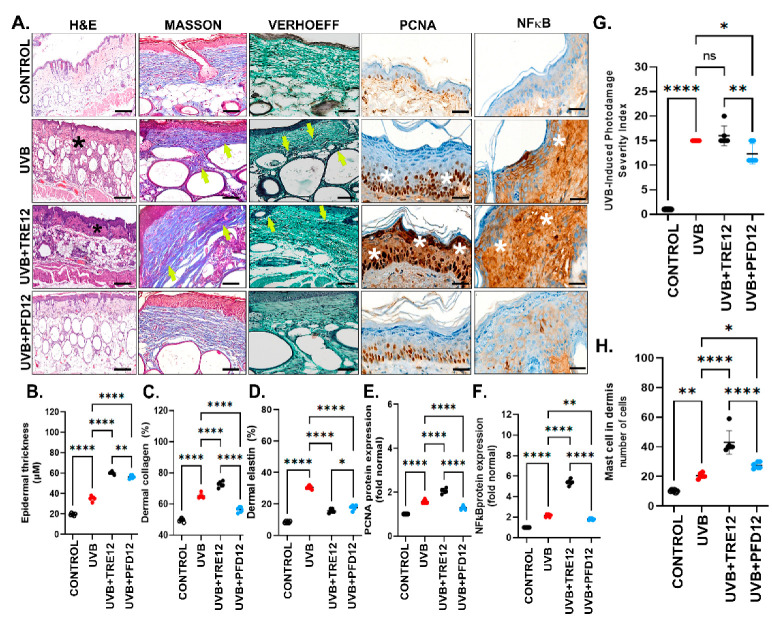
Evaluation of photodamage. (**A**) Histological analysis of photodamaged skin. Analysis included Hematoxylin and Eosin (H&E), Masson´s trichrome and Modified Verhoeff staining. Staining showed that pirfenidone treated groups display mild acanthosis and noninflammatory cells in dermis, as well as normal organization of collagen fibers (yellow arrows). (**B**) Epidermal thickness increased significantly (** *p* ≤ 0.005) in the TRE group compared to untreated mice. (**C**) Collagen fibers staining decreased in the PFD group. (**D**) PFD treatment reduced elastosis, (**E**) PFD significantly reduced PCNA quantification (*p* ≤ 0.005). (**F**) NF-kB expression was highly reduced in UVB + PFD12 mice (*p* ≤ 0.05). (**G**) Control mice reached minimal score, while tretinoin and untreated mice displayed the major scores. (**H**) Mast cells were increased in UVB + TRE12 compared to untreated UVB mice (*** *p* ≤ 0.0005) and UVB + PFD12 mice. Data are expressed as the mean ± SEM. * *p* ≤ 0.05; ** *p* ≤ 0.005; **** *p* ≤ 0.0001.

Examination of hairless mice skin exposed to UVB irradiation in H&E-stained tissue slices showed mild acanthosis and atypia of keratinocytes in the UVB group. The UVB + TRE12 group presented an increased acanthosis versus UVB + PFD12 mice (Figure 3A). Besides, keratinocyte atypia was observed only in UVB + TRE12 mice. On the other hand, the epidermal thickness of groups UVB, UVB + TRE12 and UVB + PFD12, were 34 ± 0.7 μm, 63.6 ± 0.7 μm and 53 ± 1.9 μm, respectively (Figure 3B). Finally, the UVB + TRE12 group showed a significant increase in epidermal thickness compared to the skin of UVB exposed animals. 

Regarding dermal changes, photodamage in UVB mice was evident in Masson’s trichrome staining of the dorsal skin. The evaluation found a slight increase in collagen fibers, with a compact and poorly organized parallel distribution (Figure 3A, Masson). On the other hand, collagen fibers in UVB + PFD12 group mice were more compact and showed a parallel-oriented organization of the fibers alongside the epidermis. This fact represented a hallmark of the UVB + PFD12 group, promoting an enhancing organization of collagen in photodamaged skin (Figure 3A, Masson). The Verhoeff modified staining (yellow arrows) depicted that UVB mice were absent of fibers close to the dermo-epidermal junction. These results were comparable in UVB + TRE12 mice and these findings indicated elastosis and disorganization of elastic fibers (white arrows) with accumulation in the deep dermis and modifications in the appearance of the fibers (thick and irregular) (Figure 3A, Verhoeff). The main effect of PFD in dermal changes was the improvement of elastosis. Elastic fibers were only slightly increased and distributed regularly along the dermis with a fine appearance, minor cross-linking, and recovery of the fine fibers (white arrows) in the dermo-epidermal junction. These outcomes in elastic fibers were also present in control mice (Figure 3A, Verhoeff).

Morphometric analysis indicated an increase in the percentage of dermal fibers in photodamaged skin-UVB mice which had 62.9% of collagen and 30.5% of elastin significantly higher compared to control mice. The percentage of collagen was lower in the UVB + PFD12 group (51.5%) compared to the UVB group (65.3%), and the UVB + TRE12 group (72.6%) (Figure 3C). These results support the antifibrogenic effect of PFD through collagen fibers remodeling. The percentage of elastin in UVB + TRE12 mice (14.9%) decreased significantly compared with UVB (30.5%). Nevertheless, mouse skin treated with PFD showed a significantly decrease in elastosis (17.7%), versus the UVB group (*p* < 0.05) (Figure 3D).

The anti-inflammatory effect of PFD24 in the photodamage of hairless mice exposed to UVB radiation inflammation was histologically evaluated in H&E staining. UVB mice, revealed a severe and chronic lymphocyte infiltrate with diffuse distribution (*) like TRE treated mice (Figure 3A, H&E). Interestingly, PFD12 treated mice showed only mild and diffuse lymphocytic inflammatory infiltrate (Figure 3A, H&E) suggesting an anti-inflammatory effect of PFD in photodamaged skin. 

### 2.3. PFD Regulates the Expression of PCNA and NF-κB Proteins in Mouse Skin with Photodamage

Epidermal expression of PCNA protein (Figure 3A,E) was increased in UVB mice against control mice. Otherwise, PCNA was further dramatically increased in the UVB + TRE12 group versus the UVB group (*p* = 0.0001). Noteworthy, the UVB + PFD12 group showed decreased PCNA to levels as low as the control group. On the other side, NF-κB protein expression, a master proinflammatory gene, was markedly present in epidermal cells in UVB-radiated mice which showed a two-fold increase when compared to healthy controls. Again, and remarkably, NF-κB positivity significantly increased in the UVB + TRE12 group by five-fold. As expected, UVB + PFD12 mice showed a drop in this master gene to levels compared to the UVB and UVB + TRE12 groups (Figure 3A,F). These results are consistent with the well known anti-inflammatory effect of PFD [34]. 

### 2.4. PFD Was Effective in Counteracting the Severity Index of UVB-Induced Skin Photodamage in Mice

In this study, an index based on histomorphometric parameters to evaluate mouse photodamaged-skin was conceived as described in Table 1. Epidermal, dermal, and inflammatory changes were determined with a total comprehensive score (Table 1 and Table 2) in each animal and expressed as mean ± SD per group. Non-irradiated normal skin of control mice showed a total score of 1.4 ± 0.24 points, indicating no changes in normal skin. The UVB mice group scored 15 ± 0.0 points, and UVB + TRE12 mice scored 16 ± 0.83 points denoting severe and very severe cases, respectively. The UVB + PFD12 mice group scored 12.2 ± 0.37 points with moderate harm and only one severe case, suggesting that PFD could improve photodamaged skin (Figure 3G).

The score is obtained according to the histomorphometric evaluation of the parameters of Table 1.

An increased mast cell infiltration in dermis in the UVB group was seen, suggesting a hallmark of these cells related to the effect of therapy in photodamaged mouse skin (Figure 3H). Significant differences in mast cell infiltrates were found between the control group (11.6 ± 0.67 cells/microscopic field) compared with the UVB group (17.8 ± 1.02). Although PFD12 (24.8 ± 2.2 cells/microscopic field) shows a better response than the UVB + TRE12 group (43.8 ± 4.0 cells/microscopic field), a slight significant difference is also observed against the UVB group. These findings highlight the implication of these cells in the remodeling process of dermic fibers after topical therapy. 

### 2.5. PFD Effects on Gene Expression of Inflammatory and Extracellular Matrix Molecules inthe Mouse Skin Exposed to UVB Radiation

**Figure 4:**
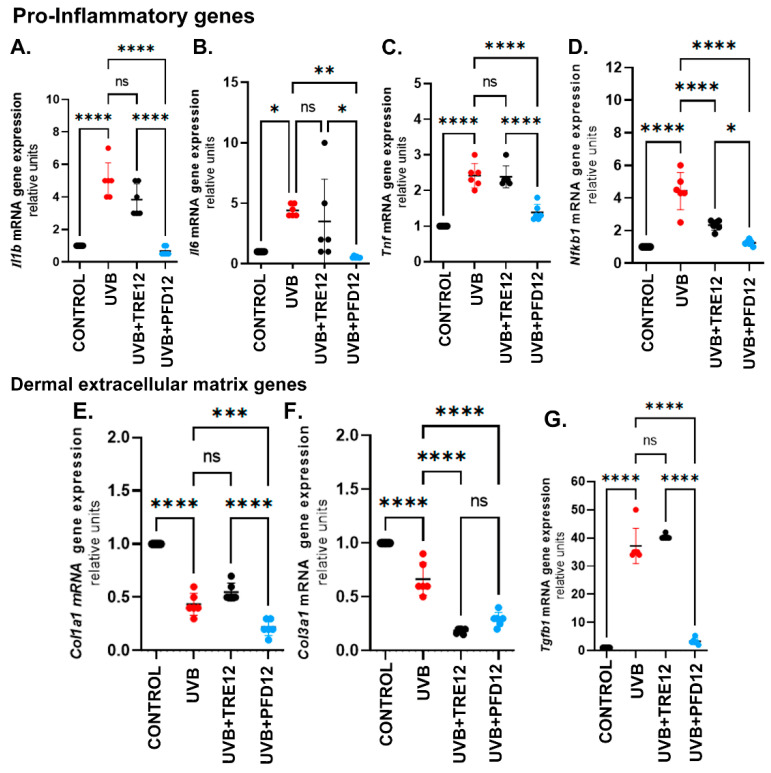
Gene expression of molecules implicated in photodamage. Pro-inflammatory cytokines (**A**) *Il1b*-1, (**B**) *Il6*, and (**C**) *Tnf* decreased after PDF treatment. (**D**) *Nfkb1* and (**E**) *Col1a1* were downregulated by PFD. (**F**) *Col3a1* mRNA levels are downregulated in UVB + TRE12 and UVB + PDF groups. (**G**) *Tgfb1* is a well-known target of pirfenidone that showed decreased expression. Data are expressed as the mean ± SEM. * *p* ≤ 0.05; **p ≤ 0.005; *** *p* ≤ 0.0005; **** *p* ≤ 0.0001.

Skin expression of proinflammatory genes linked to photodamage (*Il1b*, *Il6*, *Tnf* and *Nfkb1*) were evaluated (Figure 4A–D). The UVB group showed increased IL-1 gene expression by 4.7-folds, UVB + TRE12 by 2.7-folds, however, the UVB + PFD12 group remained like the no-damage group. Gene expression of *Il6* (Figure 4B) was upregulated in UVB mice versus the control group, on the other hand the UVB + PFD12 group showed significant responses compared with the UVB and UVB + TRE12 groups. *Tnf* (Figure 4C) was upregulated in UVB group by 2.7-folds, and UVB + TRE12 by 2.3-fold versus the control group. This response was prevented in the UVB + PFD12 group. Interestingly UVB + PFD12 mice showed significant difference compared to UVB + TRE12 mice (*p* = 0.005). The main transcription factor related with inflammation, *Nfkb1* (Figure 4D) was upregulated in UVB mice 4.3-folds, UVB + TRE12 2.0-folds and UVB + PFD12 1.8-folds vs UVB-unexposed control group. We observed significant differences between UVB untreated mice compared to UVB + TRE12 (*p* = 0.001) and PFD treated mice (*p* = 0.0001). These findings support the anti-inflammatory effect of PFD in our experimental model.

### 2.6. PFD was Effective to Regulate the Expression of Dermis Extracellular Matrix Genes 

UVB-induced photodamage impacted on dermal collagens, implicated in the process of photoaging. Gene expression of several collagens was determined. In Figure 4E, *Col1a1*was downregulated in the UVB mice group compared with the control mice (*p* = 0.0001). In the UVB + TRE12 mice *Col1a1* increased versus UVB + PFD12 (*p* < 0.0001). In addition, *Col3a1*expression was decreased in the UVB + PFD12 and UVB + TRE12 groups versus the UVB-exposed control group (*p* < 0.0001) (Figure 4F). *Tgfb1* gene expression (Figure 4G) was upregulated 31-fold in UVB mice and 39-fold in UVB + TRE12 compared to the control mice (*p* < 0.0001). A significant 30-fold downregulation of *Tgfb1*was induced by PFD in photodamaged mouse skin versus the UVB group (*p* ≤ 0.0001).

## 3. Discussion

Photodamage is a network of events triggered by signaling receptors, protein oxidation, mitochondrial and DNA damage in response to chronic UVB radiation causing histomorphologic and molecular changes on the skin. Chronic UVB radiation exposure is the most common environmental hazard associated with non-melanoma skin cancer (NMSC) type tumors [35]. Supporting the development of topical therapies that antagonize UVB skin damage, our study evaluated for the first time the anti-inflammatory, antioxidant and antifibrogenic effect of pirfenidone in mouse skin with UVB-induced photodamage.

We examined histomorphometric parameters designed by our group to analyze the severity of photodamage in untreated control animals and treated mice with PFD or TRE as anti-photodamaging agents. TRE is the most studied retinoid used for photodamage treatment, then some of the parameters evaluated have been previously described [36]. We found increased epidermal thickness in all UVB-exposed groups, a fact previously reported in TRE-treated UVB-exposed animals [20]; these data are possibly associated with the stimulation of keratinocyte proliferation factors. The lower acanthosis observed in the UVB + PFD12 group compared to the TRE group suggests a lower fibroblast proliferation due to PFD anti-inflammatory and antioxidant properties [37]. 

In the dermis, changes were mostly observed in the distribution of collagen and elastic fibers. Elastosis is the hallmark of photodamage related to aging. The use of TRE in photodamaged skin is associated with the presence of a repair zone situated in the superficial dermis, with new subepidermal collagen and displacement of the elastic fibers towards deep dermis [38]. In the group treated with PFD, improvement of elastosis was associated with the repair of damaged elastic fibers of the dermo-epidermal junction. Probably, the PFD anti-inflammatory effect observed as mild inflammatory infiltrates, is linked to less MMPs activity [39], less elastosis and improvement in normal configuration of elastic fibers. In our study, we identified a high number of mast cells infiltrates in UVB-photodamaged control skin and in UVB + TRE12 treatment, along with severe elastosis. While PFD treated mice showed a smaller number of mast cells and mild elastosis. These findings are in accordance with Kaarsen et al [40] who suggest that the mast cells might have a digesting function, since layers cleared of elastotic fibers showed few mast cells.

Collagen fibers were aligned in a parallel fashion to the epidermis and compactly organized. Morphometric analysis in Masson´s stained skins revealed an increase in the percentage of collagen fibers in the dermis of UVB-exposed control mice. This information was also described by Kligman et al. in a model of photodamage in hairless mice using UVB radiation. This fact is probably secondary to the stimulation of fibroblasts, returning to normal levels at week 10 and decreasing later by degradation secondary to late damage [39]. The anti-aging effect of retinoids can mediate the distribution of fibers maintaining collagen I and III ratio [23,41]. In our study, morphometric analysis showed a greater percentage of collagen in mice treated with TRE compared to the group treated with PFD, supporting other studies where mice skin treated with TRE led to greater amounts of collagen, elastin and fibronectin and reduced amounts of glycosaminoglycans [23,42]. Our results indicate that the PFD group showed better organization of collagen fibers. 

When gene expression of *Col1a1* mRNA was measured, decreased levels were found in all UVB-radiated mice. UVB radiation on the skin promotes the expression of MMPs through the AP-1 pathway, which also downregulates the *Col1a1* gene, contributing to a decrease in the synthesis of pro-collagen and increasing collagen degradation [43]. In addition to this, our study did not show increases in mRNA expression of *Col3a1* in mice treated with TRE or PFD.

The antifibrotic effect of PFD [34] in the photodamaged mouse skin could be due to the decreased amount of collagen, measured by morphometric analysis in Masson´s staining of UVB + PFD12 and a specific decrement in *Col1a1* mRNA gene expression. Nevertheless, histomorphometric observations confirmed a better organization of the fibers normal dermis structure. These observations made sense of the beneficial antifibrotic effect of PFD on the skin by reducing the amount and accumulation of UVB-damaged and fragmented collagen fibers in the dermis, associated with a secondary loss of the structural integrity of mice skin as previously reported [11]. Due to the fact that collagen degradation products are linked to the inhibition of new collagen synthesis [43], PFD may induce degradation of dermis collagen type I photodamaged fibers, but a dual effect of this molecule as a skin repair in photodamaged mouse skin could be supposed, as a cornerstone to generate new collagen fibers improving the organization of dermal fibers observed in UVB + PFD12 along with a healthy aspect of mouse skin observed in this group by photography (Figure 1D) and dermoscopy techniques (Figure 2C). 

On the other hand, *Tgfb1*mRNA expression was increased in UVB untreated and UVB + TRE12 mice. Previous observations suggest that excessive and prolonged TGF-β1 expression does not benefit wound healing and involves dermal changes and more severe photodamaged skin [13]. In our results, expression of *Tgfb1*mRNA was downregulated in PFD treated mice, supporting the evidence that TGF-β1 is an important target [29,30,31] and its decreased expression could be associated with less dermal damage. 

The inflammation observed in UVB + TRE12 mice was similar to UVB-radiated control mice. Since retinoids absorb light, it is known they could cause an irritant effect on the skin, developing a photochemical reaction that includes photoisomerization, generation of free radicals and lipid peroxidation [44]. This inflammatory response correlates with elevated gene expression of pro-inflammatory molecules such as *Il1b*, *Tnf* and *Nfkb1*in UVB and TRE group. This result suggests a very limited anti-inflammatory action of retinoids in UVB-exposed skin. Our results are consistent with a study from the National Toxicology Program [25] where a hairless mouse was exposed to UV-radiation and TRE 0.001%-cream was applied and severe skin irritation was developed. In contrast, in our PFD group, downregulation of pro-inflammatory molecules, particularly TNFα; suggest that PDF could act as a prophylaxis photodamage agent. In PDF treated animals lower dermal inflammatory infiltrate was observed and mild irritation was reported, evidencing tolerability and a better safety profile.

NF-κB protein expression measured by immunohistochemistry was increased in the epidermal cells of UVB and even more in TRE mice, due to the irritant effect of retinoids and their very limited anti-inflammatory effect. No previous reports evaluating this transcriptional factor in photodamaged skin treated with TRE are available. In pirfenidone treated mice, low expression of the protein and the gene remains like non-radiated control mice. Previous data from our group have demonstrated an anti-inflammatory capability of pirfenidone to regulate inflammatory cytokines via NF-κB [45]. 

DNA damage response in epidermal keratinocytes increased the expression of PCNA in suprabasal keratinocytes. Essers J et al. demonstrated that when photodamage is established, PCNA transiently accumulates at sites of DNA damage with different localization times for replication and damage repair [46]. Human and murine studies concluded that PCNA could be a marker of DNA repair and an indirect indicator of UVB-induced photodamage linked with keratinocyte atypia associated to potential of malignancy [47,48]. Different observations indicated that keratinocyte hyperplasia reverts after cessation of UVB-radiation and is associated with a lower number of PCNA-expressing keratinocytes [47]. In agreement with high PCNA expression in the UVB + TRE group compared to UVB mice, PCNA could be a hallmark of proliferation of damaged keratinocytes by TRE; associated with the tumor development observed in this group (Supplementary Materials, Figure S1) [48]. Remarkably, decreased PCNA protein expression in UVB + PFD12 mice correlates with a mild grade of epidermal and dermal photodamage observed in this group. 

In our study, skin tumors were observed in UVB + TRE12 mice. It is known that SKH-1 mice are an excellent model of photo-carcinogenesis and replicates the development of squamous cell carcinoma and other precursor lesions. One animal in the UVB + TRE12 group developed invasive squamous cell carcinoma. The association of non-melanoma skin cancers (NMSC) with the use of retinoids have been previously described in murine models [35]. However, carcinogenesis has not been reported after topical use of retinoids in humans [49], and even a clinical trial about the prevention of skin cancer by systemic administration of retinoids in humans has been performed [50]. In our results, molecular targets that could lead to carcinogenesis were increased in the TRE group, like gene expression of TGF-β1. TGF-β1 in severe photodamage has a dual effect inducing a pro-inflammatory state, and in this environment increasing growth and progress of skin tumors through proliferation of damaged epidermal cells [13]. Papillomas and keratoacanthomas-like tumors were also present in UVB + TRE12 mice (Supplementary Figure S1). Noteworthy, this type of tumor in mice has a high potential for progression to invasive squamous cell carcinoma unlike its counterpart in humans, where progression occurs in rare cases. 

An interesting proposal of our study is the evaluation of histomorphometric parameters limited by epidermal, dermal, and inflammatory changes. Significant differences using this score severity index were found in all groups of intervention compared to healthy control mice, increasing the possibility of delineating grades of severity damage. Future studies are needed to standardize this instrument to evaluate the severity of skin photodamage induced by UVB. This study is in accordance with our previous publication where we demonstrated the beneficial properties of PFD in the treatment of hypertrophic scars in children [30].

Data shown here confirm that PFD has a beneficial dual effect as an anti-fibrogenic and skin reparation drug of UVB-induced damage in dermal fibers, inducing a better alignment of collagen and elastic fibers. PFD administered since the beginning of the photodamage induced by UVB irradiation showed an anti-inflammatory effect, low expression of epidermal PCNA and the absence of skin tumors. Our data suggest that pirfenidone could be used as a protective agent against photodamage and skin tumors in a clinical scenario.

## 4. Materials and Methods

### 4.1. Mice

Protocol was approved by CUCS Research Committee, University of Guadalajara (Approbation number: C.I. 19-01). After a week of acclimatization, twenty-five male 10-weeks old SKH-1 mice were randomly allocated in groups of five animals and housed according to the Animal Care Protocol established by the Animal Facility of the Health Sciences University Center of the University of Guadalajara and the ARRIVE guidelines. Temperature of the room was 26 °C, with light and dark cycles of 12 h, and with access to balanced LabDiet^®^ 5001 Rodent Diet, and water ad libitum. 

### 4.2. UVB Photodamage Induction and Topical Therapy 

Animals were exposed to UVB radiation 3 times per week for 12 weeks to induce photodamage according to the literature [18]. A UVP^®^ 3UVTH lamp (Capitol Scientific, Austin, TX, USA) with a wavelength of 302 nm (UVB) was applied 20 cm from each animal´s back. The Schwarz & Kligman protocol was used [21]. Irradiation started with 1 Minimal Erythema Dose (MED) during the first week. This MED corresponded to 78 mJ/cm^2^, which was obtained by exposure for 2 min of radiation with the lamp. The second week radiation was increased to 2 MED for 4 min, the third week 3 MED for up to 8 min and finally, 4 MED were reached for up to 12 min, which was maintained from week 4 to week 12. Measurement of UVB energy was undertaken with an UVX Radiometer (UVP Analitik Jena US LLC, Upland, CA, USA) (Supplementary Materials, Figure S2). 

Mice were topically treated with 50 μL of TRE (0.05% Retin-A^®^ cream) or 50 μL of PFD (8% pirfenidone gel), according to each group. Treatment was applied on the dorsal surface of the mouse skin immediately after exposure to UVB radiation. The UVB control group did not receive treatment. Cream or gel absorption was allowed for 120 s. The healthy control group did not receive either UVB irradiation or topical treatment. At the end of the experimental period, mice were fasted for 4 h, and euthanized with a mix of tiletamine/zolazepam (15 mg/kg). A 2 cm^2^ portion of the photoexposed and treated dorsal skin was excised behind the neck and used for examination.

### 4.3. Histological Analysis

Tissue samples were fixed in 10% paraformaldehyde diluted in PBS, dehydrated in ethylic alcohol, and embedded in paraffin. Sections of 4 μm thickness were stained with hematoxylin-eosin (H&E), modified Verhoeff and Masson’s trichrome to evaluate inflammation, elastosis, and extracellular matrix components, respectively. Slides were scanned at 20× using the iScan Coreo v 3.4.0 equipment for complete evaluation by a pathologist blinded to the study. In addition, 10 microphotographs of the dermis at 400X were captured using an Infinity Analyze camera adapted to an Olympus microscope. Image analysis was performed using ImageJ 1.50i software. Threshold color mode was used to identify dermal fibers; data were expressed as optical density (mean ± SD) of each photograph per mouse. The degree of elastosis was assessed with a semiquantitative scale [51]. 

### 4.4. Evaluation of the Severity of Photodamage

Skin biopsies were evaluated by two certified pathologists blinded to the study using the histomorphometric parameters described in Table 1. A score was assigned to each histomorphometric parameter according to photodamage severity on a 4-degree scale (Table 2). Histomorphometric analysis used in this study was modified and validated in a previous study [51]. 

### 4.5. Immunohistochemistry

Immunostaining was performed for PCNA and NF-κB using the automated BenchMarck Ultra Ventana and the UltraView development kit. Antibodies for PCNA (ab29, Abcam) dilution 1:3000 and NF-κB (D14E12, Cell Signaling) dilution 1:600 were used. A negative control test and positive specific tissue were included as controls. Image analysis was performed.

### 4.6. Real Time Quantitative Polymerase Chain Reaction (PCR) Analysis

A portion of the skin was homogenized with TRIzol^®^ reagent. Total RNA was extracted according to the modified Chomzynsky and Sacchi technique [52]. Retrotranscription using 6 ng of total RNA was achieved using SuperScript™ reverse-transcriptase, random primers, deoxynucleotide triphosphate (dNTP), dithiothreitol (DTT) and RNAase inhibitor. Then, 2 μL of cDNA were subjected to real-time PCR using a LigthCycler 96 instrument under the following conditions: 2 min at 50 °C, 5 min at 94 °C and 45 cycles of 30 s at 94 °C and 40 s at 60 °C. Specific mouse Taqman primers and probes acquired from Applied Biosystems were used to align with *Il1b, Il6*, *Tnf*, *Nfkb1*, *Col1a1*, *Col3a1*, and *Tgfb1*mRNAs, see Table 3. Expression was normalized against 18s constitutive gene. Relative quantification using the 2-ΔΔCT method was realized by comparison to the healthy control group as an internal calibrator, gene expression levels are shown as relative expression units. 

### 4.7. Statistics 

Data are presented as the mean ± SD or standard error of the mean (SEM). A Levine test was performed to evaluate variance homogeneity. Statistical significance was determined for parametric data with one-way ANOVA and Tukey’s post-hoc test. Kruskal–Wallis and Mann–Whitney U test for non-parametric data were applied. A chi-squared test was performed for variables expressed in frequency. A *p*-value < 0.05 was considered as statistically significant.

## 5. Conclusions

Data shown here confirm that PFD has a beneficial dual effect as anti-fibrogenic and skin reparation drug of UVB-induced damage in dermal fibers, inducing a better alignment of collagen and elastic fibers. PFD administered since the beginning of the photodamage induced by UVB irradiation showed an anti-inflammatory effect, low expression of epidermal PCNA and the absence of skin tumors. Our data suggest that pirfenidone could be used as a protective agent against photodamage and skin tumors in a clinical scenario. 

## Figures and Tables

**Table 1:** Histomorphometric parameters for photodamage evaluation.

Parameter	Grade (points)
**Epidermal changes**	
Epidermal thickness *	µm
Morphology of corneous layer *	(a) Parakeratosis (b) Hyperkeratosis
Atypia of keratinocytes	(0) Absent (1) Mild (2) Moderate (3) Marked
Spongiosis	(0) Absent (1) Mild (2) Marked
**Inflammatory changes**	
Severity of inflammatory infiltrate	(0) Absent (1) Mild (2) Moderate (3) Marked
Site of inflammatory infiltrate *	(a) Perianexial (b) Perivascular (c) Diffuse (d) Mixed
Type of inflammatory infiltrate *	(a) Acute (b) Chronic (c) Mixed
Granulomes	(0) Absent (1) Present
**Dermal changes**	
Collagen fibers	Appearance (0) Normal (1) Increased (2) Compact and increased Distribution (0) Normal (1) Organized
Elastic fibers	Appearance (0) Normal (1) Mild (2) Moderate (3) Marked Elastic fibers free zone (EFFZ) (0) Absent (1) Present
Mast cells	Number for high power field (0) 0–13 cells (1) 14–26 cells (2) 27–39 cells (3) > 40 cells

* Descriptive parameters without points assigned and no association with the severity index.

**Table 2:** Photodamage Severity index.

Grade of Photodamage	Points
Mild	0–5
Moderate	6–10
Severe	11–15
Very severe	>15

**Table 3:** Assay id probes in real-time PCR.

Mouse Probe	Assay ID
*Il1b*	Mm00434228_m1
*Il6*	Mm00446190_m1
*Tnf*	Mm00443258_m1
*Nfkb1*	Mm00476361_m1
*Col1a1*	Mm00801666_g1
*Col3a1*	Mm01254476_m1
*Tgfb1*	Mm01178820_m1

## Data Availability

All data generated and analyzed during the present study are included in this published article.

## Supplementary Materials

The following supporting information can be downloaded at: https://www.mdpi.com/article/10.3390/molecules28072929/s1, Figure S1: UVB-induced skin tumors in mice treated with tretinoin (UVB + TRE 12), Figure S2: UVB exposition in hairless mice.

## References

[1] Kosmadaki M., Gilchrest B. (2004). The role of telomeres in skin aging/photoaging. Micron.

[2] D’Orazio J., Jarrett S., Amaro-Ortiz A., Scott T. (2013). UV Radiation and the Skin. Int. J. Mol. Sci..

[3] Green A., Williams G., Nèale R., Hart V., Leslie D., Parsons P., Marks G.C., Gaffney P., Battistutta D., Frost C. (1999). Daily sunscreen application and betacarotene supplementation in prevention of basal-cell and squamous-cell carcinomas of the skin: A randomised controlled trial. Lancet.

[4] Lautenschlager S., Wulf H.C., Pittelkow M.R. (2007). Photoprotection. Lancet.

[5] Tanaka K., Hasegawa J., Asamitsu K., Okamoto T. (2005). Prevention of the Ultraviolet B-Mediated Skin Photoaging by a Nuclear Factor κB Inhibitor, Parthenolide. Experiment.

[6] Rabe J.H., Mamelak A.J., McElgunn P.J., Morison W.L., Sauder D.N. (2006). Photoaging: Mechanisms and repair. J. Am. Acad. Dermatol..

[7] Talwar H.S., Griffiths C.E., Fisher G.J., Hamilton T.A., Voorhees J.J. (1995). Reduced Type I and Type III Procollagens in Photodamaged Adult Human Skin. J. Investig. Dermatol..

[8] Bissett D., Hannonand D., Orr T. (1987). AN ANIMAL MODEL OF SOLAR-AGED SKIN: HISTOLOGICAL, PHYSICAL, and VISIBLE CHANGES IN UV-IRRADIATED HAIRLESS MOUSE SKIN. Photochem. Photobiol..

[9] Fisher G.J., Datta S.C., Talwar H.S., Wang Z.-Q., Varani J., Kang S., Voorhees J.J. (1996). Molecular basis of sun-induced premature skin ageing and retinoid antagonism. Nature.

[10] Fisher J.J.V.G.J., Voorhees J.J. (1998). Molecular Mechanisms of Photoaging and its Prevention by Retinoic Acid: Ultraviolet Irradiation Induces MAP Kinase Signal Transduction Cascades that Induce Ap-1-Regulated Matrix Metalloproteinases that Degrade Human Skin In Vivo. J. Investig. Dermatol. Symp. Proc..

[11] Fisher G.J., Kang S., Varani J., Bata-Csorgo Z., Wan Y., Datta S., Voorhees J.J. (2002). Mechanisms of Photoaging and Chronological Skin Aging. Arch. Dermatol..

[12] Pittelkow M.R., Coffey R.J., Moses H.L. (1988). Keratinocytes Produce and Are Regulated by Transforming Growth Factors. Ann. New York Acad. Sci..

[13] Li A.G., Lu S.-L., Han G., Hoot K.E., Wang X.-J. (2006). Role of TGFβ in skin inflammation and carcinogenesis. Mol. Carcinog..

[14] Gallagher C., Canfield P., Greenoak G., Reeve V.E. (1984). Characterization and Histogenesis of Tumors in the Hairless Mouse Produced by Low-Dosage Incremental Ultraviolet Radiation. J. Investig. Dermatol..

[15] Wan Y.S., Wang Z.Q., Shao Y., Voorhees J.J., Fisher G.J. (2001). Ultraviolet irradiation activates PI 3-kinase/AKT survival pathway via EGF receptors in human skin in vivo. Int. J. Oncol..

[16] Rijken F., Kiekens R., Bruijnzeel P. (2005). Skin-infiltrating neutrophils following exposure to solar-simulated radiation could play an important role in photoageing of human skin. Br. J. Dermatol..

[17] Baldwin H.E., Nighland M., Kendall C., Mays D.A., Grossman R., Newburger J. (2013). 40 years of topical tretinoin use in review. J. Drugs Dermatol..

[18] Draelos Z.D., Peterson R.S. (2020). A Double-Blind, Comparative Clinical Study of Newly Formulated Retinol Serums vs Tretinoin Cream in Escalating Doses: A Method for Rapid Retinization With Minimized Irritation. J. Drugs Dermatol..

[19] Leyden J.J., Grossman R., Nighland M. (2008). Cumulative irritation potential of topical retinoid formulations. J. Drugs Dermatol..

[20] Chaqour B., Seité S., Coutant K., Fourtanier A., Borel J.-P., Bellon G. (1995). Chronic UVB- and all-trans retinoic-acid-induced qualitative and quantitative changes in hairless mouse skin. J. Photochem. Photobiol. B: Biol..

[21] Schwartz E., Sapadin A.N., Kligman L.H. (1998). Ultraviolet B radiation increases steady-state mRNA levels for cytokines and integrins in hairless mouse skin: Modulation by topical tretinoin. Arch. Dermatol. Res..

[22] Bagatin E., Gonçalves H.D.S., Sato M., Almeida L.M.C., Miot H.A. (2018). Comparable efficacy of adapalene 0.3% gel and tretinoin 0.05% cream as treatment for cutaneous photoaging. Eur. J. Dermatol..

[23] Schwartz E., Kligman L.H. (1995). Topical Tretinoin Increases the Tropoelastin and Fibronectin Content of Photoaged Hairless Mouse Skin. J. Investig. Dermatol..

[24] Griffiths C., Finkel L., Tkanfaglia M., Hamilton T., Voorhees J. (1993). An in vivo experimental model for effects of topical retinoic acid in human skin. Br. J. Dermatol..

[25] National Toxicology Program (2012). Photocarcinogenesis study of retinoic acid and retinyl palmitate [CAS Nos. 302-79-4 (All-trans-retinoic acid) and 79-81-2 (All-trans-retinyl palmitate)] in SKH-1 mice (Simulated Solar Light and Topical Application Study). Natl. Toxicol. Program Tech. Rep. Ser..

[26] Macías-Barragán J., Sandoval-Rodríguez A., Navarro-Partida J., Armendáriz-Borunda J. (2010). The multifaceted role of pirfenidone and its novel targets. Fibrogenesis Tissue Repair.

[27] Tzouvelekis A., Wolters P.J. (2018). Pirfenidone in the kaleidoscope: Reflecting mechanisms through different angles. Eur. Respir. J..

[28] Raghu G., Johnson W.C., Lockhart D., Mageto Y. (1999). Treatment of Idiopathic Pulmonary Fibrosis with a New Antifibrotic Agent, Pirfenidone. Am. J. Respir. Crit. Care Med..

[29] Armendariz-Borunda J., Islas-Carbajal M.C., Meza-Garcia E., Rincon A.R., Lucano S., Sandoval A.S., Salazar A., Berumen J., Alvarez A., Covarrubias A. (2006). A pilot study in patients with established advanced liver fibrosis using pirfenidone. Gut.

[30] Poo J.L., Torre A., Aguilar-Ramírez J.R., Cruz M., Mejía-Cuán L., Cerda E., Velázquez A., Patiño A., Ramírez-Castillo C., Cisneros L. (2020). Benefits of prolonged-release pirfenidone plus standard of care treatment in patients with advanced liver fibrosis: PROMETEO study. Hepatol. Int..

[31] Armendariz-Borunda J., Lyra-Gonzalez I., Medina-Preciado D., Gonzalez-García I., Martinez-Fong D., Miranda R.A., Magaña-Castro R., Peña-Santoyo P., Garcia-Rocha S., Bautista C.A. (2012). A Controlled Clinical Trial With Pirfenidone in the Treatment of Pathological Skin Scarring Caused by Burns in Pediatric Patients. Ann. Plast. Surg..

[32] Gasca-Lozano L.E., Lucano-Landeros S., Ruiz-Mercado H., Salazar-Montes A., Sandoval-Rodriguez A., Garcia-Bañuelos J., Santos A., Davila-Rodriguez J.R., Navarro-Partida J., Bojórquez-Sepúlveda H. (2017). Pirfenidone Accelerates Wound Healing in Chronic Diabetic Foot Ulcers: A Randomized, Double-Blind Controlled Trial. J. Diabetes Res..

[33] Rodríguez-Castellanos M., Tlacuilo-Parra A., Sánchez-Enríquez S., Vélez-Gómez E., Guevara-Gutiérrez E. (2014). Pirfenidone gel in patients with localized scleroderma: A phase II study. Thromb. Haemost..

[34] La Mora D.A.L.-D., Sanchez-Roque C., Montoya-Buelna M., Sanchez-Enriquez S., Lucano-Landeros S., Macias-Barragan J., Armendariz-Borunda J. (2015). Role and New Insights of Pirfenidone in Fibrotic Diseases. Int. J. Med Sci..

[35] Rogers H.W., Weinstock M.A., Feldman S.R., Coldiron B.M. (2015). Incidence Estimate of Nonmelanoma Skin Cancer (Keratinocyte Carcinomas) in the US Population, 2012. JAMA Dermatol..

[36] Bhawan J. (1998). Short- and long-term histologic effects of topical tretinoin on photodamaged skin. Int. J. Dermatol..

[37] Schaefer C.J., Ruhrmund D.W., Pan L., Seiwert S.D., Kossen K. (2011). Antifibrotic activities of pirfenidone in animal models. Eur. Respir. Rev..

[38] Kligman L.H., Duo C.H., Kligman A.M. (1984). Topical Retinoic Acid Enhances the Repair of Ultraviolet Damaged Dermal Connective Tissue. Connect. Tissue Res..

[39] Pillai S., Oresajo C., Hayward J. (2005). Ultraviolet radiation and skin aging: Roles of reactive oxygen species, inflammation and protease activation, and strategies for prevention of inflammation-induced matrix degradation - a review. Int. J. Cosmet. Sci..

[40] Kaarsen L.L., Poulsen T.D., de Olivarius F.F., Wulf H.C. (1995). Mast cells and elastosis in ultraviolet-irradiated hairless mice. Photodermatol. Photoimmunol. Photomed..

[41] Schwartz E., Cruickshank F.A., Mezick J.A., Kligman L.H. (1990). Topical All-Trans Retinoic Acid Stimulates Collagen Synthesis In Vivo. J. Investig. Dermatol..

[42] Callaghan T.M., Wilhelm K.-P. (2008). A review of ageing and an examination of clinical methods in the assessment of ageing skin. Part 2: Clinical perspectives and clinical methods in the evaluation of ageing skin. Int. J. Cosmet. Sci..

[43] Varani J., Spearman D., Perone P., Fligiel S.E., Datta S.C., Wang Z.Q., Shao Y., Kang S., Fisher G.J., Voorhees J.J. (2001). Inhibition of Type I Procollagen Synthesis by Damaged Collagen in Photoaged Skin and by Collagenase-Degraded Collagen in Vitro. Am. J. Pathol..

[44] Dillon J., Gaillard E.R., Bilski P., Chignell C.F., Reszka K.J. (1996). The Photochemistry of the Retinoids as Studied by Steady-State and Pulsed Methods. Photochem. Photobiol..

[45] Salazar-Montes A., Ruiz-Corro L., Sandoval-Rodriguez A., Lopez-Reyes A., Armendariz-Borunda J. (2006). Increased DNA binding activity of NF-_k_B, STAT-3, SMAD3 and AP-1 in acutely damaged liver. World J. Gastroenterol..

[46] Essers J., Theil A.F., Baldeyron C., van Cappellen W.A., Houtsmuller A.B., Kanaar R., Vermeulen W. (2005). Nuclear Dynamics of PCNA in DNA Replication and Repair. Mol. Cell. Biol..

[47] Moore J.O., Wang Y., Stebbins W.G., Gao D., Zhou X., Phelps R., Lebwohl M., Wei H. (2006). Photoprotective effect of isoflavone genistein on ultraviolet B-induced pyrimidine dimer formation and PCNA expression in human reconstituted skin and its implications in dermatology and prevention of cutaneous carcinogenesis. Carcinog..

[48] Moore J.O., Palep S.R., Salado R.N., Gao D., Wang Y., Phelps R.G., Phelps R.G., Lebwohl M.G., Wei H. (2004). Effects of ultraviolet B exposure on the expression of prolif-erating cell nuclear antigen in murine skin. Photochem Photobiol..

[49] Weinstock M.A., Bingham S.F., DiGiovanna J.J., Rizzo A.E., Marcolivio K., Hall R., Eilers D., Naylor M., Kirsner R., Kalivas J. (2012). Tretinoin and the Prevention of Keratinocyte Carcinoma (Basal and Squamous Cell Carcinoma of the Skin): A Veterans Affairs Randomized Chemoprevention Trial. J. Investig. Dermatol..

[50] Chen K., Craig J., Shumack S. (2005). Oral retinoids for the prevention of skin cancers in solid organ transplant recipients: A systematic review of randomized controlled trials. Br. J. Dermatol..

[51] Bhawan J., Olsen E., Lufrano L., Thorne E., Schwab B., Gilchrest B. (1996). Histologic evaluation of the long term effects of tretinoin on photodamaged skin. J. Dermatol. Sci..

[52] Chomczynski P., Sacchi N. (2006). The single-step method of RNA isolation by acid guanidinium thiocyanate–phenol–chloroform extraction: Twenty-something years on. Nat. Protoc..

