# Supplementary material for: Pirfenidone Protects from UVB-Induced Photodamage in Hairless Mice
Source: Molecules. 2023 Mar 24;28(7):2929. doi: 10.3390/molecules28072929 (PMC10096127; doi:10.3390/molecules28072929)
Supplement: Supplementary file 1 [file molecules-28-02929-s001.zip › molecules-2244982-supplementary.pdf]

Supplementary Materials

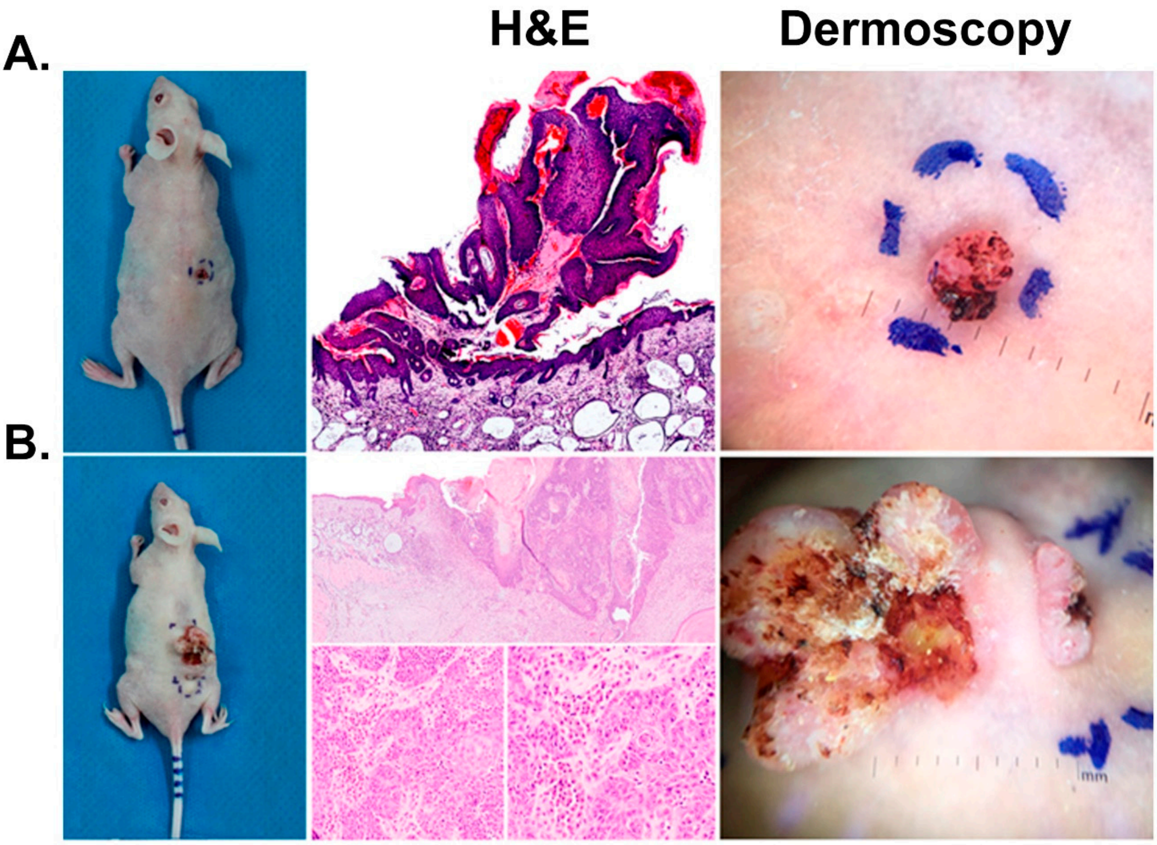

Figure S1. UVB-induced skin tumors in mice treated with tretinoin (UVB+TRE 12). Photography, histology and dermoscopy of skin tumors in mice developed during the study of 12 weeks. Both cases in TRE treated mice correspond to **(A)** papilloma, and exophitic and epidermal tumor; and **(B)** squamous cell carcinoma moderately differentiated with keratin pearls and deep infiltration with ulcerative aspect.

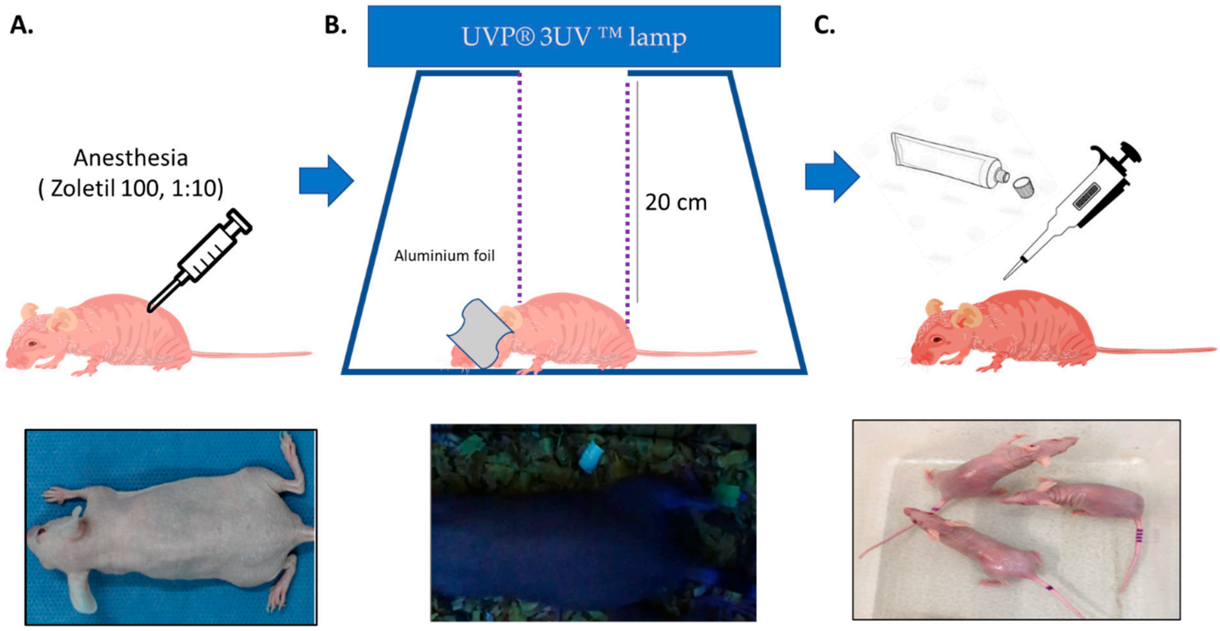

Figure S2. UVB exposition in hairless mice. A) All mice were anesthetized with 50μL of Zoletil100 (diluted 1:100)**. B)** Irradiance at the dorsal surface of the mice (20 cm from the lamp) was 0.217

mW/cm2 of UVB and was reached by exposure for 12 min as measured with an UVX Radiometer (UVP Analitik Jena US LLC). Exposure was performed three times a week, increasing one minimal erythema dose (MED) weekly until 4 MED were reached and maintained until week 12. **C**) After irradiation, 50 μL of TRE (0.05% Retin-A® cream) or 50 μL of PFD (8% pirfenidone gel) was immediately applied to dorsal surface of the mouse**.**
